# Methodologic Strategies for Quantifying Associations of Historical and Contemporary Mortgage Discrimination on Population Health Equity: A Systematic Review

**DOI:** 10.1007/s40615-024-02137-3

**Published:** 2024-09-17

**Authors:** Janelle R. Edwards, Christian Ong, Sharrelle Barber, Irene Headen, Loni P. Tabb, Anneclaire J. De Roos, Leah H. Schinasi

**Affiliations:** 1 Department of Environmental and Occupational Health, Dornsife School of Public Health, Drexel University, Philadelphia, PA USA; 2 Department of Epidemiology and Biostatistics, Dornsife School of Public Health, Drexel University, Philadelphia, PA USA; 3 Department of Community Health and Prevention, Dornsife School of Public Health, Drexel University, Philadelphia, PA USA; 4 Dornsife School of Public Health, Urban Health Collaborative, Drexel University, Philadelphia, PA USA; 5 Dornsife School of Public Health, Ubuntu Center On Racism, Global Movements, and Population Health Equity, Drexel University, Philadelphia, PA USA

**Keywords:** Mortgage discrimination, Redlining, Racial bias in mortgage lending, Homeowners Loan Corporation (HOLC), Home Mortgage Disclosure Act, Health, Built environment, Systematic review

## Abstract

**Background:**

Mortgage discrimination refers to the systematic withholding of home mortgages from minoritized groups. In recent years, there has been an increase in empirical research investigating associations of historical and contemporary mortgage discrimination on contemporary  outcomes. Investigators have used a variety of measurement methods and approaches, which may have implications for results and interpretation.

**Purpose:**

We conducted a systematic review of peer-reviewed literature that has quantified links between both historical and current mortgage discrimination with contemporary adverse environmental, social, and health outcomes. Our goals were to document the methodology used to measure and assign mortgage discrimination, to assess implications for results and interpretation, and to make recommendations for future work.

**Methods:**

Following the Preferred Reporting Items for Systematic Reviews and Meta-Analysis guidelines, literature searches were conducted in September 2022 using terms that combined concepts of mortgage discrimination, health, and neighborhood environment.

**Results:**

In total, 45 papers fit the eligibility criteria. In these, researchers investigated associations between mortgage discrimination and: (1) health outcomes (*N* = 28); (2) environmental and social exposures including heat, air pollution, greenspace, soil lead levels, and crime (*N* = 12); and (3) built environment features, including presence of retail alcohol, fast food, and tobacco stores (*N* = 5). Eleven included studies used Home Mortgage Discrimination Act (HMDA) data to identify racialized bias in mortgage discrimination or redlining, and 34 used Homeowner Loan Corporation (HOLC) maps. The construction and parametrization of mortgage discrimination or redlining and the spatial assignment of HOLC grades to contemporary addresses or neighborhoods varied substantially across studies.

**Conclusions:**

Results from our review suggest the need for careful consideration of optimal methods to analyze mortgage discrimination such as HOLC spatial assignment or HMDA index parametrization, contemplation of covariates, and place-based knowledge of the study location.

**Supplementary Information:**

The online version contains supplementary material available at 10.1007/s40615-024-02137-3.

## Background

Home ownership remains the largest financial investment for those in the lower middle class [1]. Home ownership is a gateway to wealth accumulation and financial security [2–5], and housing is a fundamental social determinant of health [6]. Historically, the U.S. federal government has aided homebuyers in making this investment, particularly during times of mass economic hardship [1]. Some of the most formative homeownership programs were implemented in the late 1930s, after the Great Depression, when the federal government aimed to revitalize the housing market by offering American families low interest mortgage repayment plans [7]. As a part of this initiative, the Federal Housing Administration (FHA) created the government-sponsored Home Owners Loan Corporation (HOLC) to, “…provide emergency relief with respect to home mortgage indebtedness, to refinance home mortgages, to extend relief to the owners occupied by them and who are unable to amortize their debt elsewhere…” [8]. As an indirect mechanism of gatekeeping mortgage loans from certain communities, HOLC lending staffers suggested that some households were more likely to default on loan payments than others. To guide decision making in loan disbursement, in 1938, HOLC lending staffers created spatially color-coded maps to illustrate lending risk, with the lending risk of neighborhoods categorized as “best,” “still desirable,” “definitely declining,” and “hazardous” and depicted in the map using the colors green, blue, yellow, and red, respectively. The red shaded communities that were termed “redlined” were predominately inhabited by African American, foreign-born, and low-income residents [9, 10]. These historical maps still serve as spatial representations of unequal investment and disinvestment, wealth accumulation and poverty, with long-lasting implications for contemporary health and well-being [11].

The discriminatory HOLC maps were used until 1968, when the federal government passed the Fair Housing Act, to protect people from discrimination when buying a home or other housing related activities. In 1975, the U.S. federal government passed the Home Mortgage Disclosure Act (HMDA), which required that select financial institutions collect and publicly disclose information on demographics and other characteristics of mortgage loan applicants, and whether the loan was granted. These two initiatives promoted greater transparency with respect to mortgage lending practices [12].

In 2016, researchers at the University of Richmond’s Mapping Inequality project digitized the HOLC color coded maps [9], which facilitated research on the links between historical mortgage discrimination and contemporary race-based environmental and health inequities. Other researchers have used data from the HMDA, accessible from the Federal Financial Institutions Examination Council’s HMDA Loan Application Register, to identify more recent mortgage discrimination or neighborhood redlining. With the resultant increased volume of research and interest in the use of these data sources, there is a need to synthesize and compare the measurement strategies and analytic approaches used, as these have important implications for results and interpretations. To our knowledge, only two systematic reviews of historical mortgage discrimination have been published [13, 14]. Both focused on redlining measured using HOLC graded maps, alone. Our review builds on these studies by including (1) more recently published papers, (2) papers that assessed redlining using HMDA data (in addition to the HOLC maps), and (3) a critical appraisal of the measurement and analytic decisions that investigators make when conducting research on redlining.

The aim of this review is to systematically identify and describe peer-reviewed studies on links between mortgage discrimination in the United States, measured with either historical HOLC maps or contemporary HMDA data, with contemporary environment, social, and health outcomes. Our primary objective is to critically describe and assess the analytic approaches authors used in assigning, assessing, and measuring mortgage discrimination using HOLC maps or HMDA data. We discuss the methods used, the conclusions that may be drawn, and make recommendations for considerations that researchers may make when planning and conducting future work on the links between mortgage loan discrimination and contemporary outcomes.

## Methods

We followed the Preferred Reporting Items for Systematic Reviews and Meta-Analyses (PRISMA) guidelines for reporting systematic reviews [15]. We searched two electronic databases (Web of Science and OVID MEDLINE) to identify eligible peer reviewed studies. Due to the unrelatability of the available Medical subject headings (MeSH) terms in OVID MEDLINE, we used text rather than MeSH search terms. MeSH terms were also not utilized in Web of Science because this search engine did not utilize this type of syntax. We searched all abstracts and titles using terms for Home Owner Loan Corporation, the Home Mortgage Disclosure Act, residential redlining. We also included database-specific search terms for both environmental and health outcomes by using the following search terms found in Supplemental Table I. The article search was first conducted between January 11th and January 14th, 2022, and then repeated on September 9th, 2022, to capture more recent publications.

### Inclusion and Exclusion Criteria

Abstracts were initially reviewed by the first author for inclusion based on whether papers quantified associations between historical redlining/contemporary mortgage discrimination and environmental/social/ or health outcomes. Articles were excluded for the following reasons, in the following hierarchy: studies that did not measure redlining or mortgage discrimination as a primary explanatory variable, review articles, theoretical commentaries, or non-peer reviewed manuscripts. The initial screening process utilized two reviewers (the first and second authors). The first author reviewed all abstracts, and the second author reviewed a random selection of 50 abstracts, in duplicate. The duplicate abstracts and decisions were compared between the two authors. The full text of articles that remained following abstract review were reviewed by two authors to determine if they met the inclusion criteria. The same exclusion criteria were used for full text review.

### Data Collection Process

The following data were extracted from the papers that met our inclusion criteria: bibliographic information including author and publication date; primary research question; primary independent variable; time period; design; study setting; population demographics (i.e., race/ethnicity, age, and sex); definition of redlining/racial bias in mortgage lending, methods used to measure and quantify redlining (data source, spatial units, reference groups, and methods used to account for missing redlining data); fully adjusted estimates of association (where applicable), and overall conclusions.

## Results

### Included Articles

**Fig. 1:**
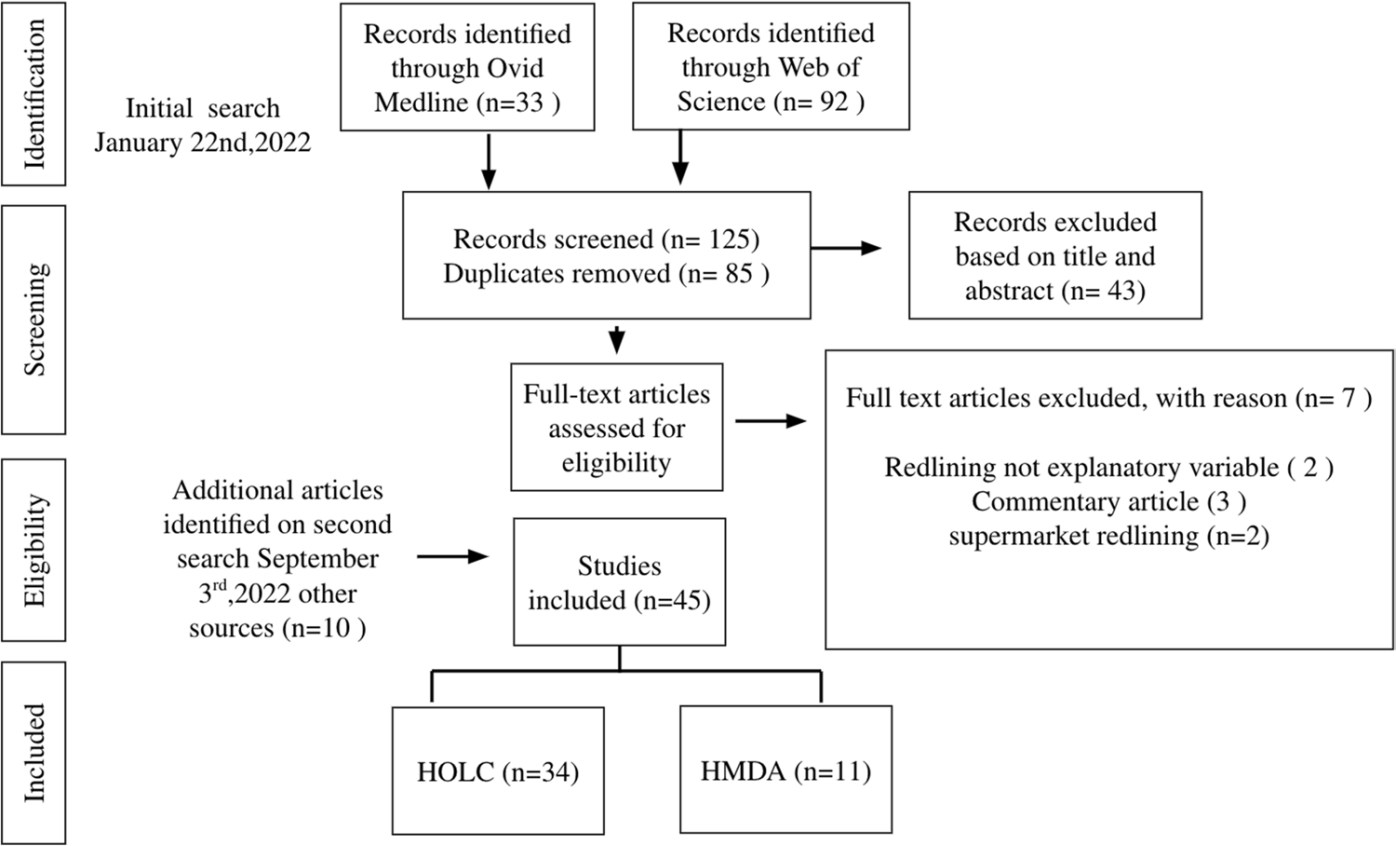
Results of search process: PRISMA flow diagram. *Abbreviations*: HOLC, Home Owners Loan Corporation; HMDA, Home Mortgage Disclosure Act

The initial search on January 22nd, 2022, in OVID MEDLINE and Web of Science yielded 85 articles, after duplicates were removed (Fig. 1). Out of this set of papers, 43 articles were excluded based on abstract review. Of the remaining 42, three were excluded because they were commentaries; two because redlining was not the primary explanatory variable; and two because they focused on restrictive covenants on retail/business locations rather than residential mortgage discrimination. An additional search on September 9th, 2022, yielded 10 more papers, all of which were retained following abstract and full-text review, yielding a total of 45 papers.

### Measures of Mortgage Discrimination and Publication Trends

**Fig. 2:**
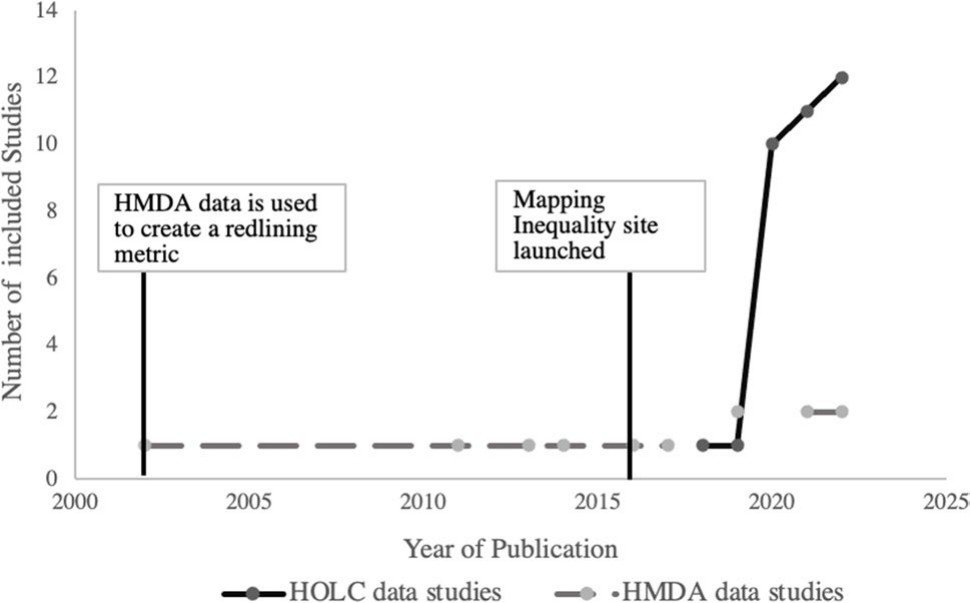
Publication years of both historical and contemporary mortgage discrimination articles included in this review. HMDA: Home Mortgage Disclosure Act; HOLC: Homeowner Loan Corporation

Of the 45 included papers, 34 utilized HOLC data and 11 utilized a HMDA-based index to characterize mortgage discrimination (we describe the details for the derivation of these measures in the sections below). An increase in the number of publications utilizing HOLC data corresponds to the time when the nationwide data were made available, in 2019, by the researchers at the University of Richmond’s Mapping Inequality project [9] (see Fig. 2). The first article that used HMDA data was published in 2002 [16], and publications using this data source remained constant through 2022.

Because the HOLC and HMDA measures represent mortgage discrimination or redlining at different points in time, and because different analytic and measurement decisions are associated with each data source, we discuss them separately below.

### Contemporary Mortgage Loan Discrimination: HMDA Data-Based Studies

#### Descriptive Summaries of Included Articles

Supplemental Table II summarizes the articles that used HMDA-based measures. These studies investigated associations with varied health outcomes, including health status and psychological symptoms [16], stress during pregnancy [18], preterm birth [18, 19], breast cancer [20–23], colorectal cancer [24], cancer related mortality [25], and HIV viral suppression [26]. While the earliest publications that derived HMDA-based measures use the term redlining, some later publications distinguish the term “redlining” from “racial bias in mortgage lending” [20]. The distinction between these terms is that “redlining” refers to the process of denying mortgages based on the underlying demographic composition of the neighborhoods to which the desired property belongs, while racial bias in mortgage lending is the process of denying mortgages based on the racial/ethnic identity of the applicants. HMDA data can be used to construct measures that correspond to each of these concepts. That is, the HMDA can be used to indicate neighborhoods: (1) where minoritized loan applicants have experienced greater bias in mortgage lending than their White counterparts, (racial bias) or (2) where applicants experienced greater denial of mortgages, when compared with other areas, based on the underlying composition of the neighborhood (redlining). From this point forward, we use language that distinguishes redlining from racial bias in mortgage lending.

### Construction of HMDA-Based Indices

All HMDA-based indices used data from the Federal Financial Institutions Examination Council’s (FFIEC’s) HMDA Loan Application Register [27]. Most studies constructed a quantitative index representing the extent of racial bias in mortgage lending within a neighborhood (i.e., racial/ethnic disparities in denial of mortgages for properties within a neighborhood). To create the index, authors [16–27], constructed multilevel logistic regression models in which the dependent variable was denial of the loan by a financial institution (coded 1/0) and the primary independent variable was race/ethnicity of the loan applicant. The logistic models also included covariates, such as the primary loan applicant’s gender and ratio of the loan amount to the applicant’s gross annual income. The resulting estimates from such models quantify the odds of mortgage loan denial for a loan applicant belonging to a minoritized racial/ethnic group, compared to White loan applications, within a defined spatial area (e.g., census tract or a spatial area defined by loan applicant characteristics), after controlling for the covariates. For example, a value of 1 would indicate no difference in loan denial between minoritized and White applicants, and a score of 1.5 would indicates that the odds of loan denial for minoritized persons was 1.5 times the odds of loan denial for White persons. Such an index can also be used to quantify odds of loan denial according to disparities in different racial/ethnic identities. For example, Mendez et al. (2013) used the index to quantify Black-White disparities in the odds of mortgage denial [17], while Gee in 2002 used calculated Asian-White disparities in the odds of mortgage denial [16]. To use the HMDA data to calculate a redlining measure that indicates the odds of mortgage loan denial within a neighborhood, authors used logistic regression models to estimate the odds of mortgage application denial for individuals inside vs. outside of designated spatial areas, with control for additional covariates, such as the primary loan applicant’s gender and ratio of the loan amount to the applicant’s gross annual income. The objective of calculating redlining in this way was to identify areas that received fewer mortgages, as compared to other areas. The resulting calculation was an index centered around 1; any value > 1 represented a neighborhood that was more likely to be denied mortgage applications than other areas, while a value < 1 indicated that the neighborhood was less likely to be denied than other areas.

### Neighborhood Definitions

The included studies used different methods to define neighborhoods. For example, Gee et al. (2002), Mendez et al. (2013), and Mendez et al. (2014) defined neighborhoods based on census tracts (the smallest spatial area provided in the HMDA data) [16–18]. Beyer (2016) and others used adaptive spatial filtering to quantify odds of mortgage loan denial within spatial areas that were independent of administrative boundaries [20, 21, 23, 24]. For their racial bias in mortgage lending measure, the authors quantified odds of denial in spatial areas (or filters) with a minimum of five Black and five White loan denials (relative to other filters meeting these threshold criteria). For their HMDA-based redlining measure, the authors quantified the odds of denial (in Black vs. White applicants) within each spatial area that had a minimum of five Black and five White loan applicants. After performing these calculations, the authors calculated the mean of the estimates within each ZIP Code Tabulation Area (ZCTA), which allowed linkage with the administrative health data being used.

### Parameterization of the HMDA-Based Indices

In models, investigators parameterized the HMDA-based measures in a variety of ways [16–18, 20, 23, 24]. Some parameterized the racial bias in mortgage discrimination measure as a continuous variable [17, 20, 24], and others created dichotomous terms, with cut-points varying across studies, from > 1.4 [16, 18] to ≥ 2 [20, 26], and ≥ 3 [23], where values above the cut-point indicated an area that experienced redlining/racial bias in mortgage lending. Similarly, authors parameterized the HMDA-based redlining measure as both continuous and as a dichotomous variable with a cut-point based on ≥ 1 to indicate a redlined area [20, 23, 24].

### Historical Mortgage Loan Discrimination: HOLC Data-Based Studies

#### Descriptive Summaries of Included Articles

Supplemental Table III provides a summary of the 34 articles that estimated redlining using HOLC maps. These studies estimated associations with a wide array of outcomes, spanning social, environmental, health, and built environment indicators. Health outcomes included COVID-19 [28]; diabetes [29]; asthma-related emergency visits [30]; cardiovascular disease [31]; adverse birth outcomes including preterm, low birth weight, and small for gestational age [32–34]; and cancers including cervical, breast, colorectal, and lung [35, 36]. Other studies estimated associations of redlining with self-rated health [37–39]. One study estimated associations of redlining with a wide array of outcomes, including prevalence of asthma, cancer, coronary heart disease, diabetes, high blood pressure, poor mental health, stroke, binge drinking, current smoking, obesity, poor sleep, health insurance status, percentage of individuals receiving a Pap smear in the past 3 years, and percentage of those with high blood pressure taking hypertension medication [40]. As for built environment outcomes, one study estimated associations with tobacco retailers [41], two with alcohol retailers [42, 43], three with food environment measures [44–46], one with heat vulnerability determinants, such as tree cover or roofing color [47], two with greenspace [48, 49], and one with locations of oil and gas wells [50]. For associations between redlining and the social environment, studies estimated associations with firearm assaults and violent crimes [51, 52], and with community resilience [53]. Lastly, other environmental outcomes included land surface temperature [54–56], flood risk [57], air pollution [58–60], and soil lead concentrations [61].

#### Spatial Assignment of HOLC Grades and Parameterization of the Redlining HOLC Grades

Table 1 shows original definitions of 1938 HOLC risk grade assignments for urban neighborhoods, as well as HOLC classification systems that have been created by researchers included in this review. Since the time that the HOLC created its risk maps, the administrative boundaries for cities, as well as small areas within cities, have changed [62]. As a result, researchers are left with challenging decisions about how to assign HOLC risk grades to neighborhoods whose boundaries have changed over time, or to neighborhoods in modern cities that were not included in historical HOLC maps. Though most authors utilized digitized HOLC maps from the University of Richmond’s Mapping Inequality project [9], this section describes the main ways by which authors chose to spatially assign historically assigned HOLC risk grades categories to contemporary neighborhood boundaries: spatial overlay, areal containment, centroid containment, and areal apportionment/areal weighting. In addition, some investigators also overlayed present-day geocoded addresses with polygons from the historical HOLC maps.

**Table 1:** Original neighborhood Homeowners Loan Corporation neighborhood HOLC risk scores and other interchangeable classifications

Original 1938 neighborhood rating definition	Color-coded map definition	Categorical redlining definition	Continuous quantitative redlining score definition
“Best”	Green	HOLC GRADE A	1
“Still desirable”	Blue	HOLC GRADE B	2
“Definitely declining”	Yellow	HOLC GRADE C	3
“Hazardous”	Red	HOLC GRADE D	4
“No rating”	Ungraded	Ungraded or GRADE E	n/a

The smallest spatial areas provided in the included HOLC studies were subregions that were smaller than census tracts [41]. Schwartz et al. in 2021 spatially overlaid digitized HOLC grades from smaller subregions that overlapped with contemporary census tracts, in addition the authors included ungraded areas in their analysis [41]. However, most studies included in this review spatially overlaid HOLC maps onto a modern setting (e.g., a city) without explicitly explaining how historically ungraded areas (“areas with no historic rating”) were assigned [46, 49, 50, 53, 56, 58, 59] and/ or did not include ungraded areas in their analyses [29, 31, 38, 44, 46–50, 54–57, 59–61].

Three studies which included sections of cities that were previously unincorporated in the late 1930s [33, 35, 36] overlaid the historical HOLC maps on current city boundaries. Areas were either: (1) fully contained by one of the 1938 HOLC map categories and thus assigned that grade; (2) “mixed,” meaning their boundaries either crossed boundaries from the historical HOLC maps or included areas without a grade from the historical maps; and (3) “other” or “ungraded” areas, meaning areas for which < 50% of the land had a HOLC grade assignment. Mixed areas, where 50% or more of the land fell into multiple HOLC grade categories were assigned the grade to which the majority of the land belonged.

Jacoby et al. (2017) [51], Wilson et al. (2020) [55], Nardone et al. (2020c) [39] spatially assigned HOLC grades by overlaying digitized HOLC maps onto the contemporary spatial unit of interest (e.g., census tract) and assigned the HOLC grade that overlapped the centroid of the contemporary unit. If the centroid landed outside a HOLC grade, then the authors either excluded the spatial unit from analysis [39] or included it but defined it as “ungraded” [51].

Areal weighting was also used as a spatial assignment technique [31, 32]. To do this, authors first assigned an increasing numerical value to each HOLC grade (A = 1, B = 2, C = 3, and D = 4). Then, weighted scores were calculated based on the proportion of the contemporary spatial unit that was covered by one or multiple HOLC grades. The resulting calculation yields a continuous score, where a higher value indicates a greater degree of redlining. For example, consider a census tract with a total area of three miles, where half aligned with a grade A area, and the other half with a grade B area; the resulting calculation for that census tract would be: [1(0.5) + 2(0.5)]/(3).

The last HOLC spatial assignment method used in the studies was utilizing present-day geocoded addresses and overlaying these with historical HOLC maps. To do this, authors [32, 47, 58] assigned geocoded units (e.g., residential properties) to the historical redlining maps. This method of spatial assignment did not require making any decisions about how to handle situations when spatial areas did not align.

#### Evaluation of Historical or Contemporary Neighborhood Sociodemographic Composition as Potential Confounders or Mediators

Some, but not all, papers [35, 42, 44, 47], adjusted for measures of contemporary potential confounders of the association between mortgage discrimination and the environmental, social, or health outcomes studied. Other papers controlled for the demographic composition that existed prior to the creation of the HOLC maps [32, 48, 51]. Jacoby et al. controlled for historical sociodemographic factors [proportion of the population who were Black, median value of owner-occupied homes (in 1940 $US), and an index of historic “concentrated disadvantage”] in their analysis [51], and Schinasi et al. (2022) [47] adjusted for an index representing 1940’s census tract level socioeconomic environment. Similarly, Nardone et al. (2020) [32] and Nardone et al. (2021) [48] controlled for sociodemographic variables, derived from the 1940 decennial census (total number of White, non-White, foreign-born White, Black, and employed residents, the number of people per housing unit, number of total homes and homes needing major repairs, the number of homes with and without refrigerators, radios, and heating, and the number of residents at different education levels) and 1940 population density in their analysis. Others adjusted for contemporary sociodemographics. For example, Trangenstein et al. in 2020 [42] and Li and Yuan in 2022 [44] controlled for current racialized residential segregation in their analysis. Furthermore, some studies measured how current demographics may lie on the causal pathway (i.e., mediate) or modify associations. For example, Krieger et al. (2020b) evaluated an indicator of racialized disadvantage (index of concentration at the extremes for racialized segregation as a potential mediator of associations between historical mortgage discrimination and cancer outcomes). In addition, two studies of food environment estimated associations with measures of gentrification (Gini index) in isolation from (and in addition to) associations with redlining and found stronger associations with the gentrification metric than with redlining—indicating that shifts in racial/economic composition within neighborhoods, over time, may have important implications for contemporary urban environments [45, 46].

## Discussion

To our knowledge, this is the largest systematic review of population health literature that has estimated associations between historical and contemporary mortgage discrimination and contemporary indicators of social, environmental, health, and well-being. Our review builds on previously published systematic reviews of redlining by (1) including an updated review of the rapidly growing literature quantifying associations between redlining or racial bias in home mortgage lending and contemporary environmental, social, and health outcomes; (2) including articles that measured mortgage discrimination using HMDA data, in addition to those using historical HOLC maps; and (3) focusing on the statistical and spatial assignment methods used. The majority of papers used HOLC map data, although some developed an index using HMDA. There was substantial variation across the papers in terms of the variables that authors adjusted for in their analyses, and whether or not investigators evaluated potential confounding or mediation by contemporary racialized residential segregation. These different methods may have important implications for the results, interpretation of the studies, and comparability across studies.

In the paragraphs below, we provide a critical appraisal of our findings and make recommendations for best practices for future research on associations between historical and contemporary mortgage discrimination and contemporary outcomes in urban settings in the United States.

## Contemporary Mortgage Loan Discrimination: HMDA Based Articles

### Strengths and Limitations of the HMDA Data Source

Eleven papers included in this review used HMDA data to assess redlining and/or racial lending bias. Strengths of the HMDA data include that it was explicitly created to elucidate unfair lending that it offers information about mortgage discrimination since 1975, and, because it is longitudinal in nature, it allows consideration of temporal trajectories in lending practices. However, the HMDA data has certain limitations. The HMDA lacks information about applicant credit score, occupation, and wealth, which could directly impact loan approval and thus confound associations. Second, because the HMDA data were first published in 1975, investigators who are interested in impacts of structural racism before that period must use alternative data sources. Third, HMDA data are only available at the census tract level, thus precluding evaluation of smaller administrative boundaries such as census blocks, or even point locations of the properties.

### Implications of the HMDA Mortgage Discrimination Definition and Recommendations

While the HMDA discrimination indices created in the studies were originally continuous, authors have often used various cut-points to create dichotomous indicators of redlining/mortgage discrimination; as a result, authors must decide what constitutes an adequate and meaningfully justifiable cut-point. These thresholds can be based on a priori hypothesis, previous literature, or be guided by distributions within the data. An important consideration, too, is the language that authors use to describe the quantitative measures derived from HMDA data. As Beyer et al. in 2016 and others [20–24] have noted, some of the quantitative indices developed using the HMDA data may better indicate racialized biases in mortgage lending, rather than redlining. The term “redlining” was generated based on the HOLC-based maps, developed in the late 1930s, which serve as spatial indicators of historic mortgage discrimination. HMDA-based indices that calculate within neighborhood differences in mortgage lending, according to the racial identity of the loan applicant, may represent a distinct—though also important—measure of racialized bias in mortgage lending. The HMDA data has also been used to construct a more contemporary measure of redlining, representing differences in lending practices according to underlying neighborhood composition. Researchers should be explicit about the concept and measurement tools that they use. We recommend that researchers develop conceptual diagrams to represent hypothesized pathways and relationships and, based on these, develop and use the quantitative measures that best represent the form of discrimination that they seek to capture.

## Historical Mortgage Loan Discrimination: HOLC-Based Articles

### Strengths and Limitations of the HOLC Data Source

A strength of the HOLC data is that it serves as a spatial indicator of historical, institutional racism and thereby provides a mechanism for quantifying impacts of this historical policy on the contemporary distribution of health and well-being indicators (including measures of the built environment). Moreover, this data source is easily accessible for major cities across the United States. However, the HOLC data comes with limitations and should be incorporated into analysis with care. Because HOLC maps provide a snapshot from the late 1930s, they do not account for dynamic changes that cities have experienced over time, such as neighborhood sociodemographic shifts (e.g., gentrification processes), and such changes could have implications for the observed associations. Secondly, because the boundaries for small-area administrative units within cities (e.g., census tracts) change over time, linking HOLC rated areas to more contemporary spatial areas can be complex. There is no consensus about how best to assign HOLC grades to administrative areas, such as census tracts, which results in decreased comparability across studies. Third, the lines used to draw boundaries for U.S. cities have changed, over time. As a result, the HOLC maps do not always align with contemporary boundaries, requiring investigators to account for previously unincorporated sections of cities. Fourth, places that never had HOLC maps drawn up for neighborhoods cannot be assessed and may be misinterpreted that these areas did not undergo structural racism in the form of redlining, yet they could have still been subjected to other racialized mortgage discriminations, such as restrictive covenants [63].

#### Spatial Assignments and Missing Data

We identified several approaches for handling the complication of historically unincorporated areas and mismatching of administrative boundaries over time. These approaches included areal weighting to compute a continuous quantitative score, using the original HOLC scores and simply excluding the historically unincorporated spatial areas, or creating categories that include “mixed” to indicate multiple HOLC grades within an administrative boundary and “unincorporated.” Each approach has its advantages and disadvantages. The simplest approach is to simply exclude all previously ungraded areas from the analysis. Advantages of this approach are its simplicity in terms of operationalization and translation and because it best reflects the historical period when the HOLC maps were created. Disadvantages of this approach are that it may lead to reduced sample size and could also result in lack of generalizability when considering a contemporary area. Areal weighting is appealing because it leads to the derivation of a continuous quantitative value. However, this approach is more complicated in terms of computation, translation, and interpretation. In 2021, researchers used areal weighting to create publicly available historical redlining scores across the United States [64]. The categorical approach that includes ungraded and mixed-grade areas enhances statistical power and may be conceptually important, as unrated areas that are near a rated area may still inherit the same HOLC rating following development. A concern of including ungraded or mixed grade areas within analyses is that they were not necessarily subject to the same disinvestments as compared to areas that experienced more substantial mortgage lending discrimination. However, in a recent publication, Noelke et al. compared different methodological approaches of authors utilizing HOLC maps and, using predictive validity tests, observed how different classifications may affect results [64]. These authors found that including “mixed” or “ungraded” areas, or spatial units that were only fractionally covered by HOLC grades, did not attenuate the relationship between HOLC grades and census tract outcomes, suggesting that these ungraded areas may be more similar to grade D than grade A areas (the referent). Furthermore, the researchers found that when including spatial units with as little as 5% of HOLC grade spatial coverage, the predictive value of the models continued to show strong performance [64]. Perhaps the best recommendation regarding spatial assignment of HOLC is to conduct sensitivity analysis utilizing various exposure assignments. For example, Mujahid et al. in 2021 utilized areal weighting for their main analysis and then spatially assigned HOLC grades by centroid containment as a secondary analysis [31]. Though Mujahid et al. found similar results between the two spatial assignment methods, consistency in results could vary depending on how geographic units changed over time in other cities. When possible, investigators may also assign geocoded point locations (e.g., residential addresses, in present-day), to the historical HOLC map polygons. In such cases, investigators do not need to deal with questions related to spatial misalignment. However, this method is not possible if the contemporary measures are assigned at the level of a polygon (e.g., tract level rates of infant mortality).

#### Adjustment for Historical or Contemporary Sociodemographic Compositional Factors

**Fig. 3:**
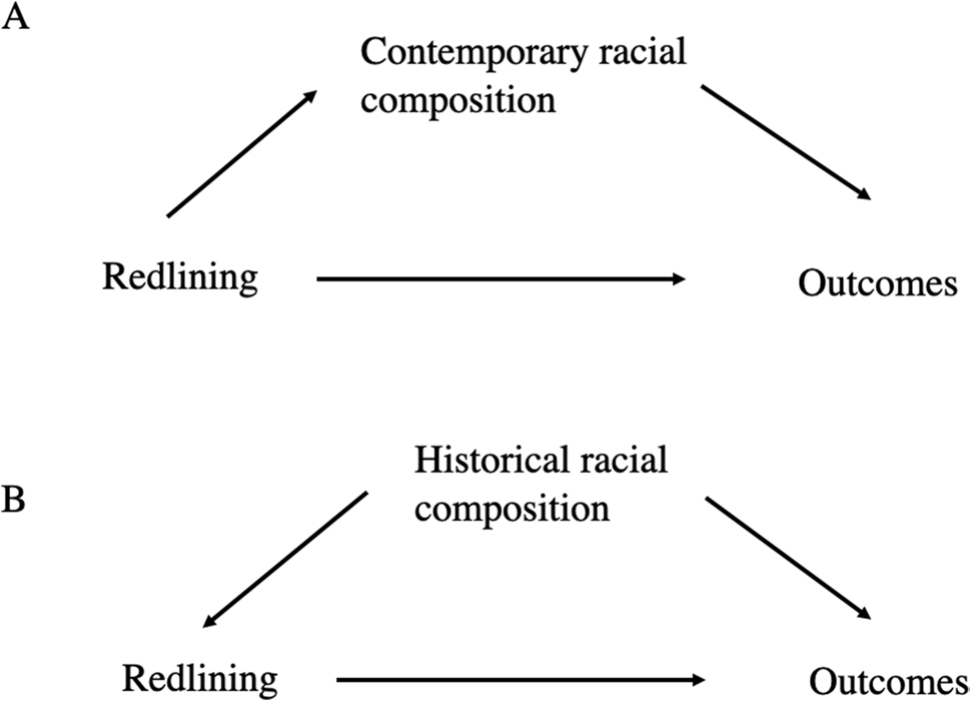
Directed acyclic graphs for the relationship between redlining, contemporary racial composition, historical racial composition, and outcome of interest

There was inconsistency across the papers with respect to how and if measures representing historical or contemporary sociodemographic composition were handled. Some adjusted for historical (before the HOLC maps were created) sociodemographic composition, arguing that this adjustment allows isolation of the impacts of the federally sponsored systematizing of biased lending practices, independent of underlying compositional factors that existed before the HOLC maps were created. However, if the HOLC grades are too closely correlated with the pre-existing compositional characteristics of a neighborhood, the adjustment could attenuate the associations or lead to model convergence issues. Some also adjusted for contemporary racialized residential segregation or sociodemographic composition [47, 51]. Arguments in favor of this approach are that adjustment for these factors allows researchers to isolate the impacts of historic mortgage discrimination practices. Arguments against this approach include the potential that contemporary racialized residential segregation or sociodemographic composition may be highly correlated with the historic HOLC grade maps, or that these factors lie on the causal pathway between the HOLC grades and the contemporary outcomes (i.e., are mediators). Figure 3 shows a conceptual diagram for relationships between redlining and outcomes. Here, panel A illustrates contemporary sociodemographic composition or racialized residential segregation as a causal intermediate between historical redlining and contemporary outcomes. To be considered a confounder, a covariate must be related to both the exposure (in this case, HOLC grade) and outcome of interest, and not lie on the causal pathway [64]. Thus, Fig. 3A suggests that contemporary racialized residential segregation or sociodemographic composition may be a causal intermediate on the pathway between historical redlining and an outcome. Thus, adjustment for a measure of contemporary racialized residential segregation may attenuate estimates of association towards the null [67]. As a result, an investigator might choose to evaluate contemporary segregation or sociodemographic composition variables, formally, as causal intermediates, through causal mediation analysis. Alternately, an investigator might adjust for these measures without formally quantifying the proportion of the associations explained by these variables. If they use this approach, investigators should interpret their results as the direct, rather than the total effect, of the relationship between redlining and the outcome, not operating through any causal intermediate variables [68, 69]. In addition, investigators my choose to evaluate effect modification and synergistic interaction between historical redlining or mortgage discrimination and contemporary compositional or segregation measures.

Panel B in Fig. 3 illustrates relationships between historical sociodemographic composition (representing the period before the HOLC redlining maps were created), historical redlining, and contemporary outcomes. Because historical composition informed the HOLC grade map and may also have shaped geographic distributions of disadvantage, irrespective of the HOLC maps, historical racial composition/demographics may confound associations of redlining and contemporary outcomes. Controlling for historical demographic variables may allow estimation of associations between HOLC grades and contemporary outcomes, independent of the composition that pre-dated the creation of the HOLC maps. However, investigators should also explore (spatial) correlation between contemporary racial composition and historical HOLC grades to ensure that the variables are not collinear. Overall, this work suggests the importance of careful consideration of relationships throughout history, and careful attention to thoughtful interpretation of results, given the covariates included in the models.

#### Place-Specific Variation and Knowledge

There is not necessarily a best-approach for all circumstances, study locations, or research questions when it comes to designing studies of links between historical redlining or biased mortgage lending and contemporary population health outcomes. It is thus critical that researchers understand the history and context of the setting in which the research is grounded. For example, HOLC ratings may have different meanings across study settings or different loan offices may have had different appraisal standards [7], and as a result, before beginning their analysis, researchers should understand the history of how HOLC grades were utilized in their study location. Authors should also know if current racial residential segregation, gentrification, or urban renewal processes are relevant to their location. They should also seek to understand the relevant historical time periods when shifts and changes within cities occurred. Though it is highly feasible to conduct large scale redlining studies across the nation with the digitized HOLC maps that are available, a potential limitation of this approach is to the challenges associated with fully understanding historical or local contexts (which could vary substantially city to city). However, multi-city studies allow investigators to evaluate differences in associations according to location, and such evaluations may have strong relevance for policy making.

### Strengths and Limitations of This Literature Review

Strengths of this review include the rigorous and comprehensive search strategy for mortgage discrimination literature. To our knowledge, this is the first review of redlining papers that includes studies of mortgage discrimination in association with health, as well as environmental outcomes. Ours is also the first to include articles that utilized HMDA data, in addition to HOLC maps. We extracted an extensive amount of detailed information on the study demographics, study design, mortgage discrimination definition or analytical approach, and results to summarize the mortgage discrimination research that has been conducted through 2022. However, we acknowledge that this review has limitations. Due to the broadness of outcomes and different redlining data sources, we were unable to utilize a study quality checklist such as the National Institutes of Health’s Quality Assessment Tool for Observational Cohort and Cross-Sectional Studies [70]. Instead, we discussed the results of this study by analytical approach of the mortgage discrimination metric, conceptualization, and/or spatial assignment.

## Conclusions and Public Health Implications

In this comprehensive systematic review of literature, we found vast heterogeneity with respect to the analytic methods and approaches used to elucidate that both current and historical mortgage lending are associated with adverse outcomes in urban U.S. settings. Additional robust, thoughtful, and carefully conceptualized research is needed to understand the longitudinal impacts of mortgage discrimination and other historically racialized policies. In future research, important factors to consider are whether the research question, analytic strategies, and mortgage discrimination metrics are appropriate to the local context, the most relevant time periods within the context of the study question, and the most appropriate spatial assignment methods for these measures within the study setting. Researchers should be explicit about the hypotheses and, based on these, carefully select the measures of bias in mortgage lending or redlining that best conform with these hypotheses. We emphasize the utility of conceptual diagrams for specifying the hypothesized longitudinal relationships and identifying appropriate covariates for the causal question, and of empirically quantifying spatial correlations across different neighborhood measures. Finally, though mortgage discrimination research focuses on macro level measurements of chronic divestment, these ecological metrics of divestment may not tell the entire story of a community. We emphasize the importance of understanding cities as dynamic entities that are prone to compositional changes. As such, researchers should develop their analyses with careful consideration for modifiers, mediators (causal intermediate), or confounders of associations, particularly between historical measures of mortgage discrimination and contemporary outcomes.

### Public Health Implications

Our review highlights the association between racialized mortgage discrimination and redlined areas with adverse contemporary outcomes, while also demonstrating how varying methodologies of exposure assessment can produce differing results. The findings of this work have significant implications for policy, emphasizing the need for researchers to exercise caution in their choice of language when framing their studies, describing their methods, and interpreting their results. Additionally, this review shows the necessity for transparent communication of these findings to stakeholders and policymakers, ensuring that the methods, data sources, and measured constructs are accurately conveyed and not misinterpreted. Decisions such as cut points in categorizing measures should be carefully considered as they can both influence and be influenced by current or former policies. It is also important to recognize that different measures and data sources, such as the HMDA and HOLC redlining maps, reflect distinct historical contexts and may thus have varied implications for policy decisions. Specifically, HMDA-based measures may reveal ongoing racialized mortgage discrimination, while HOLC-based redlining maps illustrate the lasting effects of practices established in the late 1930s. Nevertheless, policymakers should prioritize equitable urban planning by investing in historically divested communities, enhancing infrastructure, housing, and access to resources. Additionally, establishing mechanisms for monitoring and enforcing fair housing laws, including penalties for non-compliance, is vital to ensure that these laws are effectively upheld.

## Supplementary Information

Below is the link to the electronic supplementary material.

Supplementary file1 (DOCX 90.3 KB)
