# Supplementary material for: Methodologic Strategies for Quantifying Associations of Historical and Contemporary Mortgage Discrimination on Population Health Equity: A Systematic Review
Source: J Racial Ethn Health Disparities. 2024 Sep 17;12(5):3353–64. doi: 10.1007/s40615-024-02137-3 (PMC12446137; doi:10.1007/s40615-024-02137-3)
Supplement: Supplementary file 1 — Supplementary file1 (DOCX 90.3 KB) [file 40615_2024_2137_MOESM1_ESM.docx]

| **Supplement Table I.** Search terms and search strategy for each of the two databases used for this paper. | | |
| --- | --- | --- |
| Search strategy | **OVID MEDLINE** | **Web of Science** |
| 1. | (redlining OR Home Mortgage Disclosure Act OR Home Owner Loan Corporation OR Security maps OR Residential redlining) AND (Health inequities OR Health OR Adverse health outcomes) | (redlining OR Home Mortgage Disclosure Act OR Home Owner Loan Corporation OR Security maps OR Residential redlining) AND (Health inequities OR Health OR Adverse health outcomes) |
| 2. | (redlining OR Home Mortgage Disclosure Act OR Home Owner Loan Corporation OR Security maps OR Residential redlining) AND (Environmental justice OR Neighborhood resource OR Environment OR Built environment OR Climate) | (redlining OR Home Mortgage Disclosure Act OR Home Owner Loan Corporation OR Security maps OR Residential redlining) AND (Environmental justice OR Neighborhood resource OR Environment OR Built environment OR Climate) |

| **Supplemental Table II.** Characteristics of studies that quantified links between Home Mortgage Disclosure Act (HMDA)-based measures of redlining or racial bias in mortgage lending with adverse outcomes. | | | | | | | |
| --- | --- | --- | --- | --- | --- | --- | --- |
| **Author, year** | **Outcome** (s) | **Location** | **Study population and/or data source** | **Study Design** | **HMDA measure** | **Statistical methods** **to estimate associations** | **Covariates included in statistical models** **of association** |
| Beyer et al, 2016 | Survival time following breast cancer diagnosis, using breast cancer mortality or all-cause mortality as the censoring variable | Two metropolitan areas of Southeastern Wisconsin: Milwaukee and Racine | Black women living in Milwaukee and Racine who had an invasive cancer diagnosis between the years 2002-2011 | Longitudinal cohort | Redlining and racial bias in mortgage lending, estimated for a continuous surface using adaptive spatial filtering (ASF).  Indices were summarized by Zip Code Tabulation Area (ZCTA).    Racial bias in mortgage lending, was calculated from HMDA data (2004-2011) using logistic regression to create a continuous index indicating Black-White disparity in loan approval, with adjustment for sex, and ratio of the loan amount to the applicant’s gross annual income. Odds of denial were calculated for each spatial filter area defined as areas with a minimum of five denied Black applicants and five defined White applicants. Racial lending bias was parameterized as continuous and binary [<2( ref) vs. >2(biased)].    Redlining was calculated using HMDA data (2004-2011) and logistic regression models to estimate the odds of mortgage application denial for geocoded addresses inside a filter compared to addresses outside the filter, controlling for sex of the primary applicant and loan to income ratio. Index was estimated, first with omission of race/ethnicity of primary applicant, and then with inclusion. Odds of denial were calculated for each spatial filter area defined as areas with a minimum of five Black applicants and five White applicants. Redlining was parameterized as continuous and binary [<1( ref) vs. >1(redlined)]. | Cox proportional hazard regression models. | Age, stage at diagnosis, and Zip code population density |
| Beyer et al, 2019 | Black-to-White cancer mortality disparities;  Black to white disparities in cancer incidence | 100 largest US metropolitan areas. | Residents of the largest 100 US metropolitan areas, whose data gave rise to calculations of mortality counts and rates, as reported in the CDC WONDER system | Ecological | Racial bias in mortgage lending at the metropolitan statistical area level was measured using the HMDA database for 2007 through 2011, using logistic regression to create a continuous index indicating Black-White disparity in loan approval, with adjustment for sex, and ratio of the loan amount to the applicant’s gross annual income. Odds of denial were calculated for each spatial filter area defined as areas with a minimum of five denied Black applicants and five defined White applicants. Racial lending bias was parameterized as continuous. | Multivariable linear regression models to derive correlation coefficients | Socioeconomic factors (MSA median household income, proportion unemployed, and proportion without a high school diploma), Segregation (Black isolation index, Black similarity index)Models were also weighted by the size of the Black population.  To estimate whether associations between racial bias and mortgage discrimination and cancer mortality disparities could be explained by disparities in cancer incidence, calculated partial correlation coefficients that controlled for cancer incidence disparities. |
| Beyer et al, 2021 | All-cause and breast cancer–specific mortality | All metropolitan statistical areas within SEER areas in which the study cohort resided. | SEER-Medicare cohort of 27,516 women aged 66-90 years with an initial diagnosis of stage I-IV breast cancer in 2007-2009 and follow-up through 2015. The cohort was further restricted to residents in an MSA with a known census tract, and to those who did not die in the same month as diagnosis (N=27,516). | Longitudinal cohort | Redlining at the metropolitan statistical level (MSA) was estimated using logistic regression in an adaptive spatial filtering (ASF).    Redlining at the MSA level was calculated from HMDA for the years 2007 to 2013 using logistic regression to create a continuous index indicating odds of loan denial of individuals within an area versus outside an area. The index was parameterized as a four level categorical variable [<0.5 (least: ref), 0.5-1 (low), 1-2 (moderate), and > 2 (high)] | Cox proportional hazard regression models | Age, race, tumor stage, and hormone receptor (HR) status |
| Collin et al, 2021 | Breast cancer mortality, recorded through December 31, 2016 | Metropolitan Atlanta Georgia area, which included  Cobb, Clayton, DeKalb, Fulton, and Gwinnett counties | Non Hispanic Black and Non Hispanic White women aged 18 years or older diagnosed with a first primary stage I-IV breast cancer diagnosis between January 1, 2010 and December 31, 2010. Included women who resided in the metropolitan Atlanta area at the time of diagnosis.  n=8,523; recorded in the Georgia Cancer Registry. | Longitudinal cohort | Racial bias in mortgage lending at the census tract level was calculated using HMDA data for the years 2010 to 2014 using logistic regression to estimate the odds of denial of mortgage application from a non Hispanic Black applicant compared to a non Hispanic White applicant, controlling for applicant sex and the ratio of the loan amount to applicants reported annual income. to create a continuous index indicating Black-White disparity in loan approval. The index was categorized as dichotomous [<3 (ref) vs. >3 (biased)] and also modeled as a continuous index.    Redlining at the census tract level was calculated using HMDA data for the years 2010 to 2014 using logistic regression models to estimate the odds of denial of a mortgage application for a residence inside the census tract as compared to outside of the census tract. The index was categorized as a dichotomous variable [<1 (ref) vs. >1 (redlined)] and also modeled as a continuous index. | Cox Proportional Hazard regression models | Age and cancer stage |
| Gee 2002 | Self reported health status:  -Physical functioning, General health, Mental health, Global severity index, Positive symptom total, Positive symptom distress index | Los Angeles, CA | Participants from the Chinese American Psychiatric Epidemiologic Study (CAPES) n= 1503 | Cross sectional | Racial bias in mortgage lending at the census tract level was calculated using HMDA data for 1995 using logistic regression models to create a continuous index indicating Asian-white disparities in loan denial, with adjustment for applicant’s race and ratio of the loan request to applicant’s income. The index was categorized as a dichotomous variable [(non-redlined areas) > 1.4 vs ≤1.4 (ref)] | Multivariable analysis with Hierarchal linear regression modeling | Self-reported discrimination, family income, employment status, education, health insurance status, age, sex, acculturation. |
| Logan et al, 2022 | Treatment after HIV diagnoses | Multiple states across the United States | Any resident that had a positive HIV diagnosis and was linked to care within 1 month in 2017 and had a suppressed viral load in 2018 | Cross-sectional | Racial bias in mortgage lending at the metropolitan statistical areas level was calculated from HMDA for years (2014-2017) using logistic regression to create a continuous index indicating Black-White disparity in loan approval, with adjustment for sex, and ratio of the loan amount to the applicant’s gross annual income. Odds of denial were calculated for each spatial filter area defined as areas with a minimum of five denied Black applicants and five defined White applicants. The index was categorized as a dichotomous variable (<2 (ref) vs. >2) | Poisson regression with robust variance | Racial segregation, percent living below the federal poverty line, percent with less than a high school degree, median household income, ,percent without health insurance coverage, residing in a state with Medicaid, residing in a state where more than half of residents are receiving Ryan White HIV/AIDS Program, age at HIV diagnosis, sex assigned at birth, and transmission exposure route. |
| Lynch et al, 2021 | Poor physical health, poor mental health, infant mortality rate | Milwaukee, Wisconsin | n/a | Ecological | Used 2018 HMDA data for Milwaukee county to measure current lending practices; used two measures: (1) tracts with fewer than five originated loans filed per 1000 family homes, used to indicate low access to home ownership; (2) rate spread [standardized indicator of loan interest rate] used to measure high cost loans.  Also created a four-level categorical lending trajectory score, to measure perceived neighborhood lending risk over time, using the historic redlining score and 2018 lending discrimination. | Linear regression. Estimated associations of historic redlining with health outcomes, and lending trajectory and health outcomes. | none |
| Mendez et al, 2013 | Stress during pregnancy | Philadelphia, PA | A clinic-based pregnancy cohort of n = 3462 women that was gathered from a previous cross-sectional, prevalence study on chronic maternal stress and bacterial vaginosis (BV) years 1999-2004. | Cross-sectional | Racial bias in mortgage lending at the census tract level was calculated from HMDA for the years 1999 to 2004 using logistic regression to create a continuous index indicating Black-White disparity in loan approval, with adjustment for sex, and ratio of the loan amount to the applicant’s gross annual income. Odds of denial were calculated for each spatial filter area defined as areas with a minimum of five denied Black applicants and five defined White applicants. Racial lending bias was parameterized as continuous. | Multilevel (random-effects) linear regression models | race, age, marital status, education, income, previous live births, number of years living in neighborhood, the amount of sleep/rest participants reported, safety concerns and social support |
| Mendez et al, 2014 | Stress and Pre-term birth | Philadelphia, PA | A clinic-based pregnancy cohort of n = 3462 women that was gathered from a previous cross-sectional, prevalence study on chronic maternal stress and bacterial vaginosis (BV) years 1999-2004. | Cross-sectional | Racial bias in mortgage lending at the census tract level was calculated from HMDA for the years 1999 to 2004 using logistic regression to create a continuous index indicating Black-White disparity in loan approval, with adjustment for sex, and ratio of the loan amount to the applicant’s gross annual income. Odds of denial were calculated for each spatial filter area defined as areas with a minimum of five denied Black applicants and five defined White applicants.  The index categorized as a dichotomous variable [(non-redlined areas) <1.4 vs. >1.4 (redlined areas)] | Log binomial regression models | Maternal race/ ethnicity, age at interview, income, education, marital status, tobacco and alcohol usage, previous live births, housing tenure/home ownership, number of years lived in the neighborhood |
| Michaels et al, 2022 | Tract-, race/ethnicity-, and age group-specific case counts of triple‐negative and Luminal A breast cancer. 2010 population counts were used as the off-set term. | California census tracts with their centroid in a metropolitan statistical area | All non-Hispanic Black and non-Hispanic White female cases of primary breast cancer aged 20+ and diagnosed in California between January 2006 and December 2015, ascertained from the California Cancer Registry. | Cross-sectional | Racial bias in mortgage lending the census tract level was calculated from HMDA (2007–2013) using logistic regression to estimate the odds of home mortgage denial for non-Hispanic Black versus non-Hispanic White applicants, adjusting for sex and the income-to-loan ratio, to create a continuous index indicating Black-White disparity in loan approval. The index was categorized based on quintiles of the distribution (Q1 ref). | Poisson regression models with generalized estimating equations | Age and Neighborhood socioeconomic status index that compiled information on education: (median school years, percentage of high school graduates); proportion with a blue-collar job; proportion older than 16 in the workforce without a job; median household income; proportion below 200% of the poverty level; median rent; and median house value. |
| Motaba et al, 2019 | Preterm birth | Chicago, Illinois | African American mothers (n = 33,586) who gave birth between 1989–1991 | Cross-sectional | Racial bias in mortgage lending at the census tract level was calculated from HMDA for the years 1990 to 1995 using logistic regression models, adjusted for loan amount, income, and applicant gender, to estimate the odds of loan denial in Blacks versus Whites, and create a continuous index indicating Black-White disparity in loan denial. The index was categorized as a dichotomous variable [<1.4 (ref) vs. >1.4 (biased)] | Multivariable logistic regression models | Maternal age, education, marital status, parity, prenatal care and smoking. |
| Zhou et al, 2017 | All cause mortality and mortality with colorectal cancer as the cause of death. | Southeastern Wisconsin, two metropolitan areas of Milwaukee -Waukesha-West Allis and Racine | All residents with colorectal cancer diagnoses according to the Surveil- lance, Epidemiology, and End Results (SEER) | Cohort | Racial bias in mortgage lending at the zip code level was calculated from HMDA (2004-2011) using logistic regression to create a continuous index indicating Black-White disparity in loan approval, with adjustment for sex, and ratio of the loan amount to the applicant’s gross annual income. Odds of denial were calculated for each spatial filter area defined as areas with a minimum of five denied Black applicants and five defined White applicants. The index turned into a dichotomous variable [<2 (ref) vs. >2 (biased)] and kept as a continuous index.  Redlining at the zip code level was calculated from HMDA (2004-2011) using logistic regression to create a continuous index indicating odds of loan denial of individuals within an area versus outside an area. The index turned into a dichotomous variable [<1 (ref) vs. >1 (redlined)] and kept as a continuous index. | Cox proportional hazard regression models | Age, stage at diagnosis, and ZCTA population density, index of ZCTA socioeconomic status (median household income, percent, unemployed, percent renter households, percent families led by single female, and percent poverty) |
| Abbreviations: HMDA, Home Mortgage Discrimination Act; MSA, metropolitan statistical area; ZCTA, Zip code tabulation area | | | | | | | |

| **Supplemental Table III.** Characteristics of studies that quantified links between adverse outcomes and redlining using Homeowners Loan Corporation (HOLC) map data | | | | | | | |
| --- | --- | --- | --- | --- | --- | --- | --- |
| **Author, year** | **Outcome** | **Location** | **Study population and/or data source** | **Study Design** | **Methods used to assess exposure to redlining** | **Statistical methods** **to estimate associations** | **Covariates included in statistical models** **of association** |
| Asher et al, 2021 | US Census Bureau’s 11-factor Community Resilience Estimates (CRE); | Columbus, Ohio | Community Resilience estimates in Columbus census tracts | Ecological | Spatial (union) overlay of digitized grades (A-D) on current CT, ungraded areas were included | Spatial descriptive analyses, and t- test | None |
| Benns et al, 2020 | Counts of gunshot victims within original HOLC neighborhoods | 310 neighborhood areas for Jefferson County, Kentucky | All gunshot victims residing in Jefferson County, KY from 2012 to 2018;  Homicides, accidental shootings, police shootings, and those with unknown circumstances were excluded. | Ecological | Counts of gunshot victims were interpolated into A-D grade CB group from the original HOLC using areal weighting maps.  In primary analyses, gunshot victims residing in ungraded areas were not analyzed. However, ungraded areas were included in sensitivity analyses. | Zero inflated Negative binomial regressions with spatial random effects | Percentage of the block groups that were non-Hispanic Black, Hispanic, aged 15–24, in poverty, percentage of block housing that is vacant, population density, total area of block group, and the total population within each block group. |
| Fortner et al, 2021 | Soil lead concentration ; housing variables (housing vacancy density, median home values tax delinquency) | Springfield, Ohio | Community partnerships in Springfield Ohio census tracts | Cross-sectional | Boundaries from the HOLC maps were used to assign  number of vacancies per square mile, median home sale  value, tax delinquency rate, and lead soil concentration, within each HOLC assigned neighborhood area.  No mention of how mixed grades were assigned, but ungraded areas were not included in the reporting of the analysis results. | Descriptive analyses | None |
| Gonzalez et al, 2022 | Presence of oil and gas wells | 33 U.S. cities across the United States where urban oil and gas wells were drilled and operated | Census tracts across United states | Cross-sectional | The geographic unit of observation was the HOLC-graded neighborhood.  Assessed cumulative exposure to all oil, gas, oil and gas, injection, or unknown wells sited at any time within 100 m of the neighborhood boundary.  Also assessed exposure to wells drilled before and HOLC appraisal occurred in each city. | Analysis of variance and target maximum likelihood estimation | Propensity score matching was used to control for confounding by identifying neighborhoods with different grades but that were comparable in terms of sociodemographic factors.  For the subset of neighborhoods with apportioned 1940s census data, estimated propensity scores using 1940s sociodemographic characteristics (total population; proportion of Black, foreign-born, and  non-White employed residents; proportion of residents who had  completed high school; number of homes; median value homes;  proportion of homes in need of major repair; proportion of homes with  radios; and number of people in the home)., |
| Hoffman et al, 2020 | Summertime intra-urban land surface temperature anomalies (compared with the area-wide mean land surface temperature)  Impervious surface land cover  Tree canopy cover | 108 US cities or urban areas | Studied zones by US census Bureau region: Northeast (n=26), South (n=29), Midwest (n=41), and Western (n=12). | Ecological, cross-sectional | HOLC maps were overlayed with Landsat 8 imagery tiles. Zonal Statistics in ESRI’s ArcGIS Spatial Analyst toolbox were used to estimate the mean of the derived Landsat 8 land surface temperatures within each individual HOLC security rating polygon, and the temperature anomaly from the area-wide mean land surface temperature.  Overlapping HOLC grades were treated as two distinct polygons or merged into one based on the discretion of the authors. Ungraded areas were not included in analysis. | post-hoc ANOVA multiple comparisons test | None |
| Hollenbach et al, 2021 | Preterm birth, peri viable birth | Rochester, NY and 8 counties of Monroe County, New York (Finger Lakes region of NY) | Participants came from a retrospective cohort of patients with live births from 2005 to 2018 in the Finger Lakes Region perinatal and obstetric data system | Cross-sectional | Spatial overlay of digitized grades on zip codes.  Mixed grades were included in analysis, although zip codes with three or more historic designations were included separately in statistical analyses. | Multivariable logistic regression with random effects for zip code to account for within-zip code correlations | Modern sociodemographic (poverty levels, and educational attainment) maternal and paternal race |
| Huang and Sehgal, 2022 | Built environment/ health outcomes (life expectancy, teen birthrate, percentage births at term, percentage of babies born with satisfactory weight and percentage children testing positive for elevated blood lead levels. age- differentiated mortality, percentage mothers receiving prenatal care, and per population rate of liquor stores, fast food restaurants in CSA | Baltimore, Maryland | The population of Baltimore, MD | Ecological/Spatial | Spatial overlay of digitized grades (A-D) on planning board-defined community statistical areas (CSA). Largest HOLC category by area within a CSA was then taken to represent the CSA’s HOLC categorization. | Multivariable ordinary least squares regression | Current day sociodemographic characteristics (neighborhood median household income and proportion African America) |
| Hyrcyna et al, 2022 | The Air pollutant, nitrogen dioxide (NO2) | Ten most populated metropolitan areas in the Midwest (11 cities) | Population in St Paul, Minneapolis, Cleveland, Milwaukee, Omaha, Detroit, Wichita, Columbus, Chicago, Indianapolis, and Greater Kansa City. | Ecological/Spatial | NO2 values were bounded and average spatially by the HOLC polygons.  Unclear how they assigned mixed grades; ungraded areas were not included in analysis. | Descriptive statistics | None |
| Jocaby et al, 2017 | Counts of: (1) firearm assaults and (2) violent crimes that occurred in Philadelphia, 2013-2014. | Philadelphia, PA | Population of Philadelphia | Ecological/Spatial | Spatial overlay of digitized grades on 2010 CBs.  Blocks were assigned to one of five categories (green, blue, yellow, red, or not zoned) based on corresponding centroid. Then 2010 CBs were assigned to 1940 census tracts based on location of internal centroid. | Conditional autoregressive Poisson regression models, with present day population as an offset term. | 1940 demographics( proportion Black, median value of owner occupied homes, index of concentrated disadvantage)  Concentrated disadvantage calculated as sum of: proportion of the population aged 25+ without a high school certificate, proportion of homes that were renter occupied, proportion of homes without a radio, proportion or homes without a mechanical refrigerator, proportion of homes without central heating, proportion of homes with more than one person per room.  As a secondary analysis, replaced 1940 census measures with corresponding measures from five year estimates of the American Community Survey (2014) |
| Kreiger et al, 2020a | Preterm Birth | New York City, NY | Women who had preterm (singleton) births from 2013 to 2017 | Cross-sectional | Overlaid HOLC maps and assigned color-coded risk categories (A–D).. “Mixed” CT were tracts whose boundaries crossed multiple HOLC area boundaries or included areas without a grade. Mixed grade tracts were assigned the HOLC grade into which at least 50% of land fell. Tracts with 50% or more of land that was not in any HOLC grade were coded as “other.” | Logistic regression analyses; Multilevel GEE | Model adjusted for maternal race/ethnicity,  age at giving birth, nativity, educational level.  Also adjusted for the index of concentration of extremes measuring the extent to which tracts contained high concentrations of high income White households versus low income Black households. |
| Kreiger et al 2020b | Diagnosis for primary invasive cervical, breast, lung, and colorectal cancer | 28 municipalities in Massachusetts with digitized redlining maps | Men and women with primary invasive cancer diagnosed between January 1, 2001, and December 31, 2015.  Four cancer types: breast, cervix, colorectal, lung. | Cross- sectional | Assigned a HOLC grade to census tracts based on percentage of land area included in the HOLC map area. “Mixed” census tracts were tracts whose boundaries crossed multiple HOLC area boundaries or included areas without a grade. Mixed grade tracts were assigned the HOLC grade into which at least 50% of land fell. Tracts with 50% or more of land that was not in any HOLC grade were coded as “no grade assigned.” | Multivariable Poisson regression allowing for overdispersion analysis, without outcome data aggregated for each calendar year into strata by cases’ census tract of residence at the time of diagnosis, race/ethnicity, and sex/gender also conducted a formal mediation analysis | Controlled for Age at diagnosis, sex/ gender, race/ethnicity as potential confounders; evaluated five measures of social spatial polarization, quantified using the index of concentration of extremes, for: income polarization, racial privilege, racialized economic segregation, housing tenure. The ICE measures were evaluated formally as a potential mediator |
| Lane et al, 2022 | Air pollution, concentrations of NO2 and PM2.5 | National, 202 cities across the United States | Population across United States | Ecological | Used georeferenced 1930s era HOLC and then linked HOLC maps to individual U.S. Census blocks 2010.  Did not include ungraded areas in analysis. | Population weighted statistics | None |
| Lee et al, 2020 | Off-premise alcohol outlets | Northern California cities | Population in California Cities (Oakland, Berkeley, Albany, Emeryville, Alameda, and Piedmont) | Ecological | Spatial overlay of digitized grades (A-D) on census block groups. Calculated areal proportions of each census block area that fell within each of the four HOLC classes, grades were assigned based on the highest HOLC grade in the census block.  Ungraded areas were included in analysis. | Bayesian spatial Poisson regression models | Local and adjacent population, median household income |
| Li and Yuan, 2021 | Cumulative daily COVID-19 infection cases and COVID-19 deaths | New York City | Population in New York City | Ecological | Spatial overlay of digitized grades (A-D) on Zip Code Tabulation Areas (ZCTA).  Calculated areal proportions of each ZCTA that fell within each of the four HOLC classes, grades were assigned based on the highest HOLC grade in the zip code.  Ungraded areas were not included in the analysis. | Two-level mixed effect linear regression models | Proportion of population >65, proportion with lower than high school degree, household median income, household size, Covid-19 test rate, number of days tracked |
| Li and Yuan, 2022 | Food environment | 102 urban areas across the United States | Population across United States | Ecological | Spatial overlay of digitized grades (A-D) on census tracts and then calculated the land area that a certain grade occupied in each CT and computed its percentage (area occupied by the grade/ total land area in the census tract).  Ungraded areas included in analysis. | Hurdle models with random effects | CT sociodemographic characteristics and population density, city-level racial segregation and natural log of the population |
| Linde et al, 2022, | Diabetes mortality and years of life lost | Seattle, Washington | Population in Seattle, Washington | Ecological | Spatial overlay of digitized grades (A-D) on census tracts. Calculated areal proportions of each census tract that fell within each of the four HOLC classes to create a continuous score. Also explored binary comparisons HOLC A/B versus HOLC C/D.  Ungraded areas were not included in analysis. | Spatial autoregressive regression | None |
| Linscott et al, 2022 | Flood risk social factors | Nashville, Tennessee | Population in Nashville, Tennessee | Ecological | Spatial overlay of digitized grades (A-D) on census tracts. No specifics on how spatial alignment was assessed.  Ungraded areas not included in spatial descriptive analysis | Spatial descriptive | None |
| Lynch et al, 2021 | Poor physical health, poor mental health, infant mortality rate | Milwaukee, Wisconsin | Population in Milwaukee, Wisconsin | Ecological | Spatial overlay of digitized grades (A-D) on census tracts.  Assigned grades A-D a numerical value (1-4), and calculated weighted historic redlining scores for each census tract by calculating the proportion of HOLC grades contained within each boundary. Continuous scores ranged from 0.5 to 4, where 4 corresponded to the highest degree of redlining.  Excluded tracts with more than 50% of area not assigned a grade.  Treated redlining scores as continuous in analysis.  Ungraded areas not included in analysis | Linear regression. Estimated associations of historic redlining with health outcomes, and lending trajectory and health outcomes. | None |
| McClure et al, 2019 | Self-rated health trajectories | Detroit, MI | Population-representative sample of Detroit residents, derived from the Detroit neighborhood health study | Cross- sectional | Digitized and linked the historic map with a current map (A-D) of Detroit, then drew polygons around the areas deemed (redlined, grade D) and calculated their spatial area in square meters, this resulted in a continuous measure where D was a 1 on the scale and intersection of other grades (A-C) lowered the score | Linear regressions | None |
| Mujahid et al, 2021 | Ideal cardiovascular health, ideal cardiovascular health behaviors, and ideal cardiovascular health factors. | Six sites across the United States (Baltimore City and County, MD; Chicago, IL; Forsyth County, NC; Los Angeles County, CA; New York City, NY; and St. Paul, MN) | Participants aged 45 to 84 and self-identified as non-Hispanic White, non-Hispanic Black, Hispanic, and non-Hispanic Chinese were gathered from Multi-Ethnic Study of Atherosclerosis (MESA). Included participants of the MESA neighborhoods ancillary study and those living outside HOLC map areas. | Cross-sectional | Spatial overlay of digitized grades (A-D) on census tracts  Assigned HOLC based on Areal appointment by defining grades A-D with a numerical value (1-4) and then summed scores based on land area.  Ungraded areas were excluded from analysis.    Conducted sensitivity analyses using other exposure assignment methods (based on assigning the grade to which the census tract centroid fell, and based on proportion of land area that falls into a census tract. | two-level hierarchical linear models, which accounted for participants nested within census tracts, stratified by racial/ethnic subgroups. | Models adjust for age, sex, education, and income. |
| Nardone et al, 2020a | Birth outcomes (preterm, low birth weight, small for gestational age, perinatal mortality)  Secondary outcomes were very preterm, very low birth weight, neonatal mortality | California (Los Angeles, Oakland, and San Francisco) | All births occurring in California between January 1, 2006 and December 31, 2015 | Cross-sectional | Spatial overlay of digitized grades (A-D) on points of geocoded addresses.  Ungraded areas not included in analysis. | Logistic regression models, weighted using propensity score weights | 1940 CT sociodemographic characteristics (total number of White, non-White, foreign born White, Black, employed residents, number of people per housing unit, number of total homes and homes needing major repairs, number of homes with and without refrigerators, radios and heating, number of residents at different education levels) and historical 1940’s population density, with adjustment made using propensity scores |
| Nardone et al 2020b | Rates of asthma emergency visits | 8 cities in California | Population in 8 cities in California (Fresno, Los Angeles, Oakland, Sacramento, San Diego, San Francisco, San Jose, Stockton). | Ecological | Every census tract was assigned a HOLC risk grade by superimposing CT centroids onto HOLC security maps. | ordinary least squares regression with natural log-transformed visit rates | Diesel exhaust, average annual ambient PM2.5 concentration, poverty rate, city random effect |
| Nardone et al 2020c | Prevalence of 14 health indicators, divided into three categories.  Seven health outcomes:  prevalence of asthma, cancer, coronary  heart disease, diabetes, high blood pressure, poor  mental health, and stroke.  Four unhealthy behaviors:  Binge drinking, current smoking, obesity, and  poor sleep.  Three preventative measures:  health insurance status, percentage of individuals receiving  a Pap smear in the past 3 years, and percentage of  those with high blood pressure who are taking hypertension  medication | 9 cities across the United States | Population in 9 cities (Atlanta, Chicago, Cleveland, Los Angeles, Miami, New York, Oakland, San Francisco,  and St. Louis). | Ecological | Every census tract was assigned a HOLC risk grade by superimposing CT centroids onto HOLC security maps. CTs were excluded if centroids landed outside of HOLC Security Map boundaries. | Descriptive analyses, mapping, and correlation analysis | None |
| Nardone et al, 2021 | Greenspace (Summer and annual average normalized difference vegetation index [NDVI]) | 239 cities across the United States using data from all cities with HOLC maps | Population in 239 cities across the United States. | Ecological | Superimposed NDVI raster images onto HOLC shapefiles and extracted mean NDVI value for each HOLC polygon. Cell values for each HOLC polygon were weighted based on proportion of spatial overlap with a 1940s census tract and normalized to account for imperfect overlap of NDVI pixels with HOLC polygons.  Ungraded areas not included in analysis. Made comparisons with adjacent grades (B vs. A, C vs. B, and D vs. C) | Targeted maximum likelihood estimation using propensity scores and weights | 1940 CT sociodemographic characteristics ( percentage non-White residents, Black residents, foreign-born residents, number of people per unit, median home value, percentage of homes needing major repairs, percentage of employed residents, per- centage of homes without a radio, percentage of residents who completed high school), 1940 population density, ecoregion, Census region |
| Nowak et al, 2022 | Proportion of tree cover and impervious surfaces, Forest ecosystem service values (Air pollution removal and associated health impacts; annual carbon sequestration by trees; avoided runoff) | Across the United States | n/a | Ecological | HOLC maps were combined with 2011 National Land Cover Database 30 meter resolution tree cover and impervious cover maps to quantify the amount and percent tree and impervious within each redlining class by place. NLCD tree and impervious surface estimates were compared with random sampling of aerial images from 2011 using Google earth.  Ecosystem service values were calculated for each redlining risk score in each place.  Did not include ungraded areas in analysis. | Descriptive; calculated and compared differences in percentage cover by HOLC risk grade class | None |
| Sadler et al, 2021 | Healthy food availability index | Baltimore, Maryland | Population in Baltimore | Ecological | Spatial overlay of digitized grades (A-D) with residential parcels.  Ungraded areas were excluded from analysis | Multivariate regression | Socioeconomic distress index at the census block group level (based on low educational attainment, living below the poverty line, unemployment, and lone parent families); percent of African American population for each census block group; age of the residence of each parcel, blockbusting gentrification |
| Saverino et al, 2021 | Mean temperature | Richmond, Virginia | Population in Richmond | Ecological | Spatial overlay of digitized grades (A-D) on census blocks. The highest proportion within each census block was calculated and assigned to a HOLC grade.  Ungraded areas were not included in analysis, comparison were A/B combined and C/D combined | Quartile analyses | None |
| Schinasi et al, 2022 | Heat vulnerability characteristics (tree canopy cover, street trees, dark roof, flat roof, flat dark roof) | Philadelphia, PA | Population in Philadelphia | Ecological | Randomly selected 100 latitude/longitude points corresponding to residential properties from within each of the following categories of HOLC grade neighborhoods: A, B, C, D.  Ungraded areas not included in analysis. | Modified Poisson regression generalized estimating equation models with an exchangeable correlation matrix models | 1940s census tract level socioeconomic environment (estimated using an index that combined) and racial segregation (estimated using the index of concentration at the extremes for race),  present-day measures of racialized economic deprivation was evaluated in a secondary analysis |
| Schwartz et al, 2021 | Tobacco retailer density | 13 cities in Ohio | Population across Ohio | Ecological | Spatial overlay of digitized grades (A-D) on smaller subregions of each 1930 HOLC graded area that overlapped with a part of a 2016 census tract. The subregions served as the geographic unit of analysis.  Ungraded areas (Grade E) included in analysis, pairwise comparisons. | Negative binomial models with generalized estimating equations (GEE) to account for spatial dependence over subregions. | CT sociodemographic characteristics (race/ethnicity, poverty, age, and population size) city fixed effect. |
| Shaker et al, 2022 | Low income low grocery store access | Census tracts in the continental US located within areas originally graded by HOLC | Population across the United States | Ecological | Spatial overlay of digitized grades (A-D) on census tracts. Every CT was assigned a HOLC risk grade by superimposing census tract centroids onto HOLC security maps. CTs were excluded if centroids landed outside of HOLC Security Map boundaries. | Logistic regression models with generalized estimating equations with a robust covariance estimate | Sociodemographic (Proportion Black, Proportion Asian, Proportion Hispanic, Proportion other race, Proportion with no automobile, Proportion disability, Proportion under 5 years, proportion over 75 years, median age of housing, Gini Index, Population density). |
| Trangenstein et al, 2020 | Clusters of alcohol outlets | Baltimore, Maryland | Population in Baltimore | Ecological | Spatial overlay of digitized grades (A-D) on census blocks.  No specifics on how spatial alignment was assessed.  Ungraded areas included in analysis | Multiple (multivariable) logistic regression | ICE, Concentrated disadvantage index, retail land use location quotient, convenience stores, schools, drug arrest density, vacant housing density, population density, percent single households |
| Wenzel and Schyler, 2022 | Neighborhood emission of CO, filterable PM2.5, SO2, VOCs;  Uncontrolled and/or severe asthma; secondary asthma related outcomes. | Pittsburgh/Allegheny County, Pennsylvania | Participants from University of Pittsburgh Asthma & Environmental Lung Health Institute@UPMC asthma focused research registry | Cross-sectional | Buffered polygon based approach with proximity analysis was used to assign geocoded addresses of registry participants and stationary or point emission sources of industrial/commercial air pollutants to being located within or nearby one of the historical HOLC neighborhoods.  Generated HOLC grade emission estimates, and PM2.5 emissions/modeled ambient levels by summing emissions by each HOLC grade.  Generated asthma related outcome estimates by aggregating across HOLC grade. | Spearman’s rho correlations with post-hoc Dunn tests or Cochran-Armitage tests for trend used to assess monotonicity/asymmetry across HOLC grades | None |
| Wilson, 2020 | Land surface temperature | Baltimore Maryland, Dallas Texas, and Kansas City Missouri | Population in Baltimore, Dallas and Kansas City | Ecological | Spatial overlay of digitized grades (A-D) on Landsat 8 images Assigned HOLC grades based of internal centroid . Ungraded areas not included in analysis. | Descriptive statistics including Box plots | None |
| Wright et al, 2022 | Incidence of primary invasive breast cancer, overall and by tumor estrogen (ER + , ER −) and progesterone (PR + , PR −) receptor status | 28 municipalities in Massachusetts | All residents of Massachusetts living in 28 municipalities with HOLC maps diagnosed with incident breast cancer between January 1, 2005 and December 31, 2015 and categorized as female by the cancer registry, and the corresponding total population of female residents in MA. | Longitudinal cohort | Assigned a HOLC grade to census tracts based on percentage of land area included in the HOLC map area. “Mixed” census tracts were tracts whose boundaries crossed multiple HOLC area boundaries or included areas without a grade. Mixed grade tracts were assigned the HOLC grade into which at least 50% of land fell. Tracts with 50% or more of land that was not in any HOLC grade were coded as “no grade assigned.” | Multilevel Poisson regression | Racialized group membership,  incidence rates were age standardized |
| Abbreviations: HOLC, Homeowner Loan Corporation; MSA, metropolitan statistical area; ZCTA, Zip code tabulation area; CT, census tract; CB, census block group. | | | | | | | |
